# Age-dependent effects of the β_3_ adrenoceptor agonist CL316,243 on human and rat detrusor muscle strips

**DOI:** 10.1007/s00424-023-02877-x

**Published:** 2023-11-22

**Authors:** Charlotte Petereit, Katrin Porath, Simone Rackow, Karoline Kernig, Oliver W. Hakenberg, Rüdiger Köhling, Timo Kirschstein

**Affiliations:** 1 Oscar Langendorff Institute of Physiology, University of Rostock, Gertrudenstrasse 9, 18057 Rostock, Germany; 2 Department of Urology, University of Rostock, Rostock, Germany; 3 Center of Transdisciplinary Neurosciences Rostock (CTNR), University of Rostock, Rostock, Germany

**Keywords:** Patients, Isometric force, Potassium, Beta3, Antibody, DAPI

## Abstract

Motility of detrusor smooth muscle includes adrenergic relaxation and cholinergic contraction. Since the latter may be deregulated in overactive bladder (OAB) pathophysiology, anticholinergics are the standard therapy but occasionally less tolerated due to side effects such as dry mouth and constipation. β_3_ adrenoceptor agonists also alleviate OAB symptoms by relaxing the detrusor muscle. Their age dependence, however, is far from understood. To address this issue, we induced contractions with KCl (60 mM) and carbachol (from 10 nM to 100 μM) in the presence of the β_3_ adrenoceptor agonist CL316,243 (from 0.1 to 10 μM) in both human and rat muscle strips. Our results confirmed that both contractions were attenuated by β_3_ adrenoceptor activation in both species, but with differing age dependence. In humans, specimens from mid-life subjects showed a significantly more pronounced effect of CL316,243 in attenuating carbachol-induced contractions than those from aged subjects (Cohen’s d of maximal attenuation: 1.82 in mid-life versus 0.13 in aged) without altering EC_50_. Conversely, attenuation of KCl responses by CL316,243 increased during ageing (Spearman correlation coefficient = -0.584, *P*<0.01). In rats, both KCl- and carbachol-induced contractions were significantly more attenuated by CL316,243 in samples from adolescent as compared to aged samples. Immunohistochemistry in human detrusor sections proved β_3_ adrenoreceptor abundance to remain unaltered during ageing. In conclusion, our findings suggest differential age-dependent changes in human β_3_ adrenoceptor-dependent attenuation of detrusor contraction in terms of electromechanical versus pharmacomechanical coupling; they may help understand the differential responsiveness of OAB patients to β_3_ agents.

## Introduction

Overactive bladder (OAB) is a clinical syndrome composed of urinary urgency, increased micturition frequency, and nocturia, with or without urge incontinence [1] affecting 10–17% of the adult population in Japan, Europe, and North America [24, 33]. Standard antimuscarinic therapy allows for the intended bladder relaxation but entails some unfavourable side effects such as dry mouth and constipation owing to muscarinic receptors expressed throughout the parasympathetic nerve system [16]. Novel compounds with M_3_ receptor subtype specificity made no exception to this, since side effects were mostly linked to M_3_ receptors, too. Hence, up to 90% of OAB patients discontinue antimuscarinic medication within 12 months [8, 42, 45]. Consequently, research has set out to discover alternative routes of alleviating OAB symptoms. One major achievement consisted of detecting the β_3_ adrenoceptor as the preponderant sympathetic receptor subtype in the human bladder [20, 22, 27, 39, 50, 51, 56], but see [52]. Subsequently, observing that β_3_-adrenergic compounds relieved the overactive bladder [48] fostered the development of mirabegron as the first β_3_ adrenoceptor agonist approved for OAB [9].

Pharmacological treatment of OAB needs to respect the issue of receptor expression changes during normal bladder ageing, which potentially interferes with the effectiveness of drugs. In fact, some studies demonstrated age-related reduced functional responsiveness of β adrenoceptors in both rats [14, 18, 38] and humans [27]. In particular, the β_2/3_ adrenoceptor agonist BRL37,344 possessed less efficacy in detrusor samples from both aged rats [18] and elderly humans [27]. These data probably imply that reduced responsiveness to β_3_ adrenoceptor activation could be a feature of normal ageing. In line with this, Li et al. suggested there were a diminished number of binding sites in aged bladder tissue [27], but Niclauß and colleagues found the radioligand [^3^H]-dihydroalprenolol used in that study to be unsuitable for β_3_ adrenoceptors [37]. Moreover, immunostaining using β_3_-specific antibodies revealed sustained expression of all β adrenoceptors throughout the bladder with increasing age [28]. Beyond that, ageing mice even harboured enhanced β_3_ adrenoceptor levels, at least on the transcriptional level [13]. Thus, while OAB treatment with β_3_-adrenergic compounds has become clinical reality, its mechanism is far from understood, and the issue of age-dependent efficacy and tolerability was only recently addressed [36].

Based on the current knowledge, we still lack a comprehensive picture of β_3_ adrenoceptor expression and function across species and, more importantly, during ageing. Here, we aimed to study the functional responsiveness of human and rat detrusor smooth muscle strips to the highly selective β_3_ adrenoceptor agonist CL316,243 [4, 6, 59] towards high KCl and carbachol challenge. Whereas CL316,243 responsiveness consistently decreased in rats during ageing, human tissue revealed this compound to affect high KCl and carbachol responses differentially and age-dependently. Since β_3_ adrenoceptor immunoreactivity in humans was not altered during ageing, we conclude that intracellular cascades may be responsible for these effects.

## Materials and methods

### Preparation of human detrusor samples

Human detrusor samples were obtained from 21 patients (69 ± 9 years old, mean ± SD, range 55 to 85 years, median 70 years, 12 males, nine females). All in vitro experiments in this study using human material were approved by the local ethics committee (University Medicine of Rostock), and the informed consent to participate in this study was obtained from each patient. Since it was essential to get fresh tissue directly cooled within the operation theatre, we pursued the following approach: Once informed of an upcoming sample, we provided a beaker with 4 °C cold HEPES-buffered and calcium-reduced storage solution containing (in mM) 145 NaCl, 4.5 KCl, 1.2 NaH_2_PO_4_, 0.1 CaCl_2_, 1.0 MgSO_4_, 0.025 Na_2_-EDTA, and 5 HEPES (pH=7.4). This solution had been proven to show pH stability for at least 1 day. During surgery (radical cystectomy due to urothelium carcinoma), a tissue sample was excised from the macroscopically unaffected wall of the detrusor muscle (roughly 2 cm in width). This sample was immediately submerged into the storage solution of the above composition and transferred to the laboratory. Then, the detrusor samples were freed from mucosal and adipose tissue and cut into 4–8 muscle strips of some 1 cm length and 3 mm width. Subsequently, nylon threads were sutured to these specimens to fix them later in an organ bath chamber at 37 °C (Panlab ML0146/C, ADInstruments, Spechbach, Germany) filled with bicarbonate-buffered and normal-calcium solution that contained (in mM) 120 NaCl, 4.7 KCl, 2.5 CaCl_2_, 1.2 MgCl_2_, 30 NaHCO_3_, 1.2 KH_2_PO_4_, 0.5 Na_2_-EDTA, 5.5 glucose, 2 sodium pyruvate (pH=7.4), and was gassed with carbogen (95% O_2_ and 5% CO_2_). Since each organ bath contained four chambers (with 25 ml each), we used one or two organ baths for human bladder experiments. The whole procedure from bladder removal to insertion in the organ bath chamber lasted less than 3 h.

### Preparation of rat bladder samples

Rat bladder specimens were obtained from 112 Wistar rats (RRID: RGD_737929, 43 males and 69 females, minimum 40 days, first quartile 58 days, median 70 days, third quartile 137 days, maximum 560 days, Charles-River, Sulzfeld, Germany). Rats were decapitated in deep anaesthesia with diethylether, and the bladder was quickly removed and submerged in the 4 °C cold HEPES-buffered and calcium-reduced storage solution of the above composition. Each bladder was cut into four strips (some 5 mm length in and 1 mm in width) to fix them thereafter in an organ bath chamber as described before for human detrusor strips (i.e. all four specimens into one organ bath). To ensure a preferably atraumatic preparation, we did not remove the mucosa from rat bladder tissue. The whole procedure from bladder removal to insertion in the organ bath chamber lasted less than 30 min.

### Isometric contractions and relaxations in vitro

Human and rat specimens were carefully inserted into the organ bath without any tension. Once submerged in an organ bath chamber, the specimen was exposed to buoyant force and body temperature (37 °C). After roughly 10 min, we slightly stretched the specimens (~2 mN) to ensure that the force transducers (Panlab MLT0201), coupled with bridge amplifiers (Panlab ML224) and connected to an analogue-to-digital converter (PowerLab 4/30, LabChart 7, ADInstruments), were sufficiently sensitive to record both isometric contractions and relaxations. From our previous studies [25, 32] we learned that human tissue often needed prolonged equilibration periods of several hours to produce robust and reproducible KCl responses. Therefore, equilibration time was >30 min in rat and >6 h in human tissue. Since intra-surgical excision procedures and sample transport conditions showed a higher variability in human than in rat tissue, experiments with human tissue were carried out only in specimens developing a stable baseline tone (113 out of 123, baseline tone 3.9 ± 1.8 mN, mean ± SD, *n*=113).

We always used the following protocol: At the beginning, the specimens were challenged with 60 mM KCl for 10 min (termed *K*_pre_) to obtain a robust isometric contraction by adding either 500 μl of 3 M or 1000 μl of 1.5 M stock solution (i.e. dilution 1:50 or 1:25, respectively) to the organ bath (25 ml). We did not compensate for the concomitant hypertonicity, for smooth muscle cells do not contract under these conditions [26]. After washout, specimens were allowed to recover for 30–40 min before assessment of the effect of the β_3_ adrenoceptor agonist CL316,243 (referred to as CL hereafter, obtained from Tocris, UK, cat. no. 1499), because it was proven to be more selective towards β_3_ adrenoceptors than the clinically used compound mirabegron (selectivity >10,000-fold, [15]), which was also true for human cell lines [4, 59]. To this end, we applied 25 μl of CL stock solution (0.1, 1, or 10 mM) into the organ bath chamber to yield the final CL concentrations of 0.1, 1, or 10 μM (dilution 1:1000). Following 10 min of incubation with CL, 60 mM KCl (10 min) was added to the organ bath as before (termed *K*_CL_). After 30–40 min recovery, specimens were challenged with 60 mM KCl (10 min) to test for CL washout (i.e. *K*_post_). Since these experiments, however, revealed that CL showed incomplete washout, we did not alternate CL and vehicle tests in the same specimen. Rather, we carried out parallel time control experiments, and a given chamber was either dedicated to being a CL or a time control experiment. Since specimen size as well as orientation of smooth muscle cells affect contractile force, we analysed KCl responses in a paired condition (i.e. without/before versus with/after CL).

Having assessed the effect of CL on KCl-induced contraction, we tested up to five different concentrations of carbachol (referred to as CCh hereafter, ranging from 10^−8^ to 10^−4^ M in a randomised fashion), each lasting for 10 min, by adding 25 μl of the respective stock solution (dilution 1:1000) into the organ bath chamber to obtain a concentration-response curve (CRC). However, concentrations greater than 10^−6^ were suspected to render further CCh testing impossible owing to desensitisation. In these cases, only three different concentrations were analysed (i.e. stopping the experiment after the highest concentration of >10^−6^ M). Each CCh-induced response was normalised to the KCl response of the very specimen to produce a CCh CRC either in the presence or absence of CL. In addition, we performed Boltzmann fits by minimising the sum of squared residuals (i.e. the sum of the squared difference between observed and fitted values using the EXCEL solver.xlam add-in) to yield the median effective CCh concentration (EC_50_) as well as the maximum effect (E_max_).

### Immunohistochemistry of human β_3_ adrenoceptors

Human detrusor samples (*n*=10 patients) were fixed in 0.1 M phosphate-buffered saline (PBS) containing 4% paraformaldehyde for at least 1 day, cryoprotected in 30% sucrose for 3 to 5 days, frozen in 2-methylbutane, and then stored at −80 °C. Horizontal sections (14 μm) were cut on a cryostat and stored (−80 °C). For immunohistochemistry (IHC), heat-induced antigen retrieval (10 min cooking time, 0.05% Tween-20 in 10 mM citrate buffer, pH 6.0) was carried out to enhance the immunofluorescent signal. After cooling down for 20 min and 3×10 min washing in PBS, sections were first incubated for 20 min with 0.1% triton-X (in PBS), washed with PBS (2×10 min), and then incubated for 60 min with 5% normal goat serum (NGS). Sections were then allowed to incubate with the primary antibody (rabbit anti-β_3_ 1:200 in 1% NGS/PBS, Alomone AAR-017, RRID: AB_2039720 [46]) at 4 °C overnight. Following 3×10 min washes with PBS, the second antibody (Cy3 goat anti-rabbit, RRID: AB_2534029, 1:200 in 1% NGS/PBS) was allowed to bind, before sections were embedded with ProLong^TM^ Gold Antifade Mountant with DNA stain DAPI (P36931, Thermo Fisher Scientific). To avoid investigator bias in IHC studies, one experimenter (T.K.) chose the specimens, while another experimenter (S.R.) who was blinded regarding age performed both staining and analysis of IHC experiments including selection of viewing fields.

### Statistical analysis

All contractions and relaxations were assessed as absolute force data (in mN) as well as percentages of the first KCl response (referred to as %*K*_pre_). All data in the manuscript are given as mean values ± standard deviation (SD). Prior to statistical evaluation, all technical replicates from the same animal were converted into one median per sample. Then, data were analysed using the Mann-Whitney *U* test, Wilcoxon signed rank test, or two-way analysis of variance with post-hoc tests (for concentration-response curves) using SigmaStat 3.5 software. Owing to the exploratory nature of the study, we did not perform power analysis beforehand to calculate sample sizes, and *P* values are deemed descriptive. Statistically significant differences were indicated by asterisks (*P<0.05, **P<0.01) in all figures.

## Results

### The β_3_ agonist CL316,243 dampens human detrusor contraction in an age-dependent manner

**Fig. 1:**
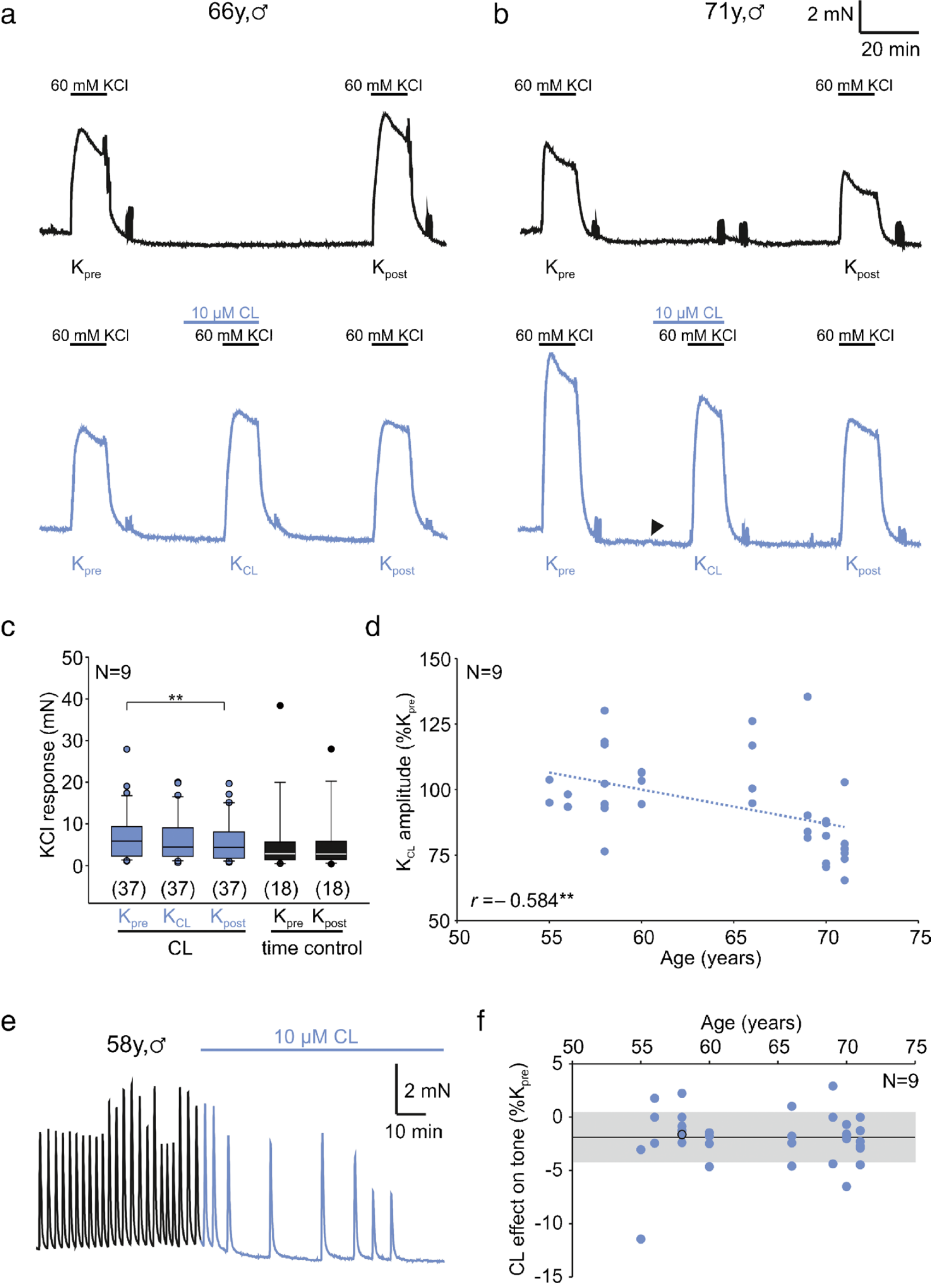
CL316,243 inhibits KCl-induced contraction in human detrusor. **a, b** KCl-induced contractions in a 66-year-old patient (**a**) and a 71-year-old patient (**b**). Upper traces (black) demonstrate reproducible KCl-induced contractions (termed *K*_pre_ and *K*_post_). Lower traces (blue) show three subsequent KCl-induced contractions; the second one was recorded in the presence of CL316,243 (termed *K*_CL_). Note that *K*_CL_ is not reduced in the 71-year-old patient, but in the 66-year-old patient. **c** The KCl response under CL (*K*_CL_) was not different from *K*_pre_, but *K*_post_ was significantly different from *K*_pre_ (Wilcoxon signed rank test). In time control experiments, no significant differences were found. *n*-numbers in brackets refer to muscle strips, *N*-numbers refer to patients. **d** The *K*_CL_ amplitude was negatively correlated to age. **e** Representative trace showing the acute CL effect on baseline tone and spontaneous activity. **f** The CL effect on baseline tone was significant, but without statistically significant age dependence. The black circle indicates the subject of **e**

Aiming to explore the role of the β_3_ adrenoceptor in human detrusor muscle, we first evoked isometric contractions by 60 mM KCl in this tissue with or without the specific β_3_ adrenoceptor agonist CL316,243 (CL hereafter). Figure 1a shows a representative trace of two subsequent KCl-induced contractions (black traces, termed *K*_pre_ and *K*_post_) recorded from a specimen from a 66-year-old patient, which demonstrate their reproducibility. In an adjacent chamber of the organ bath, we tested a second specimen from the same patient for its responsiveness of KCl-induced contractions towards pre-applied CL (10 μM). In this patient, however, CL failed to depress the KCl response (blue traces in Fig. 1a; termed *K*_CL_). On the other hand, detrusor specimens from a 71-year-old patient also showed reproducible KCl responses (*K*_pre_ and *K*_post_ in Fig. 1b), but here CL reduced its amplitude (blue traces in Fig. 1b; termed *K*_CL_), and, in addition, the KCl response after CL washout (*K*_post_) remained smaller when compared with that at the beginning of the experiment (*K*_pre_). In a total of 37 specimens taken from nine patients, CL failed to significantly affect the subsequent KCl response (*K*_CL_), but reduced *K*_post_ after CL washout (*P*<0.01, Wilcoxon signed rank test; blue box-whisker plots in Fig. 1c). In contrast, there was no such drop of the KCl response in parallel time control experiments (*n*=18 specimens from nine patients, black box-whisker plots in Fig. 1c). Since we wondered whether this statistically significant, but rather small effect could be relevant, we correlated the patients’ age to the relative size of *K*_CL_ (Fig. 1d), which left us with a statistically significant negative correlation (Spearman correlation coefficient = −0.584, *P*<0.01; Fig. 1d). This result prompted us to analyse the CL effect in greater detail. In fact, this compound regularly reduced baseline tone (see arrowhead in Fig. 1b). Moreover, out of 37 specimens, four (from three patients) presented spontaneous activity before the application of CL: in all these cases, CL abolished spontaneous activity (see example in Fig. 1e). On average, activation of β_3_ adrenoceptors significantly depressed the baseline tone by 0.13 ± 0.13 mN or 1.8 ± 2.4% of *K*_pre_ (*n*=37, *P*<0.01, Wilcoxon signed rank test), which, however, showed no statistically significant correlation to ageing (Spearman correlation coefficient = 0.211, *P*=0.121; Fig. 1f).

Since equilibration time in human tissue was up to 15 h, the question arose whether this could have affected contractility. To address this issue, we plotted the first mean KCl response (i.e. one value per subject) to the equilibration time and found no statistically significant correlation (Pearson correlation coefficient = 0.0598, *n*=17 patients, *P*=0.343). Furthermore, we quantified the baseline tone at the beginning and at the end of the prolonged equilibration phase showing a small but statistically significant reduction (−0.74 ± 1.5 mN, mean ± SD, *n*=113). To exclude that this reduction could have contaminated the KCl response under CL, we separately calculated the baseline tone reduction for specimens that were to be challenged later with CL (−0.57 ± 0.97 mN, *n*=52) versus those which were to serve later as controls (−0.88 ± 1.89 mN, *n*=61). These analyses demonstrated that the specimens of the CL group were even slightly more stable than those serving as controls. We therefore think it unlikely that the prolonged equilibration in human tissue could have affected the observed CL effects on contraction.

**Fig. 2:**
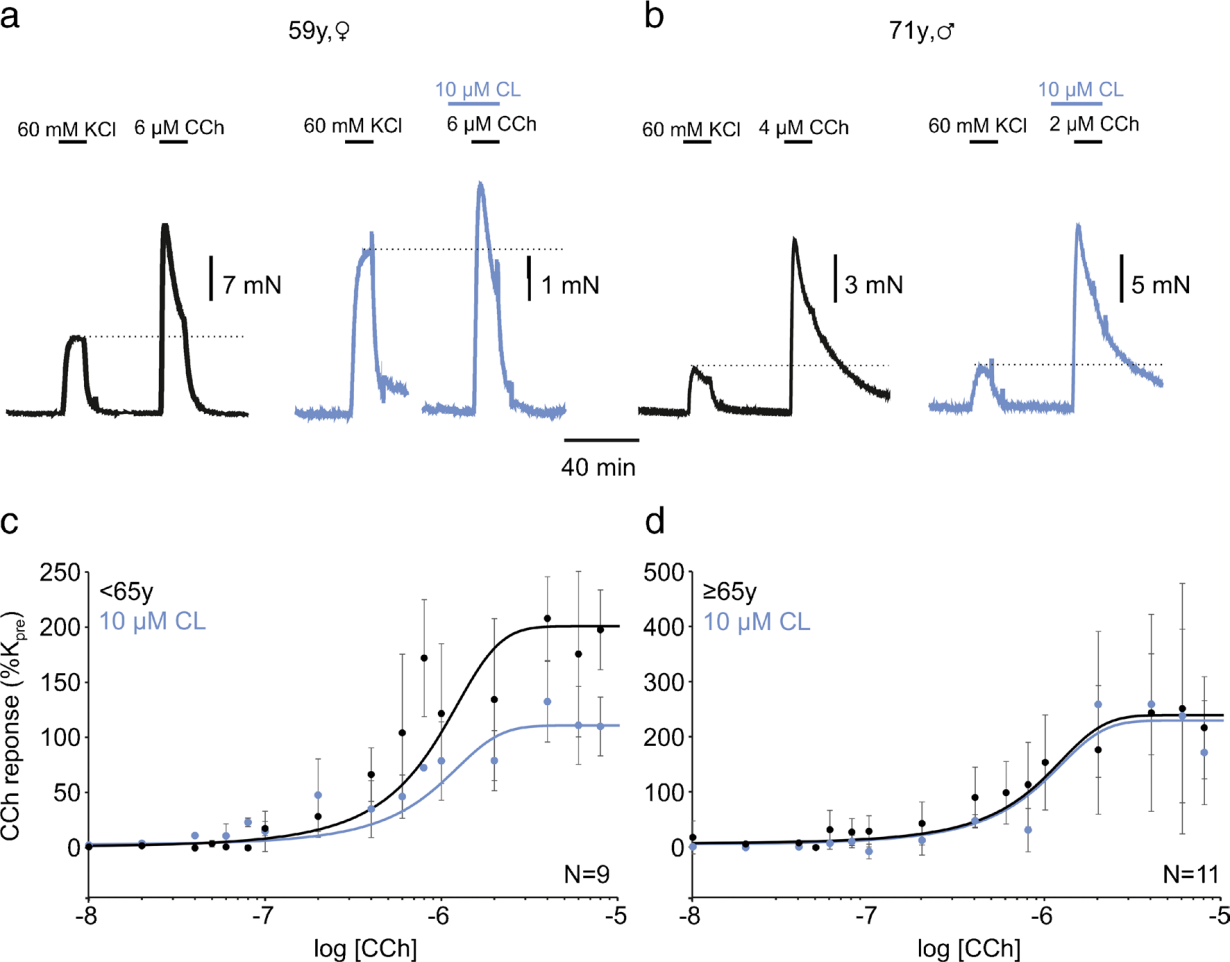
CL316,243 inhibits CCh-induced contraction in human detrusor. **a, b** CCh-induced contractions were normalised to KCl-induced contractions (indicated by dotted lines). In a 59-year-old patient (**a**), administration of CL (blue traces) markedly reduced the CCh-response in comparison to the KCl response. In a 71-year-old patient (**b**), CCh responses were hardly affected by prior CCh administration. **c** Concentration-response curve of CCh in patients <65 years with (blue) and without (black) prior CL application. Data are presented as mean ± SD (*n*=1–11), *N-*number refers to patients. **d** Concentration-response curve of CCh in patients ≥65 years with (blue) and without (black) prior CL application. Data are presented as mean ± SD (*n*=1–23), *N*-number refers to patients

Next, we asked whether the β_3_ adrenoceptor agonist CL also affects CCh-induced contractions in human detrusor tissue. To control for systematic effects of strip size on the CCh response, we normalised this CCh response to *K*_pre_ of the same specimen and obtained CRCs with and without CL. A representative example of this experiment (Fig. 2a) shows the traces of a specimen that exhibited a KCl response of around 12 mN and a CCh response of about 30 mN (i.e. 250% *K*_pre_; black traces). When applying CL prior to CCh in another specimen from this 59-year-old patient, CCh elicited a reduced response (140% of *K*_pre_; blue traces in Fig. 2a) indicating a clear β_3_ adrenoceptor-dependent attenuation of the CCh response. In a specimen from a 71-year-old patient, however, the CCh response was unresponsive to the CL pretreatment (roughly 350% with or without CL; black and blue traces in Fig. 2b, respectively). To address the ageing effect statistically, we divided our patient cohort into two sub-cohorts of about the same sample size, i.e. either below 65 years (referred to as mid-life) or above (referred to as aged). In the first sub-cohort, the Boltzmann fit revealed the native CRC (i.e. without CL, black symbols in Fig. 2c) with an EC_50_ of 0.8 μM and a maximum effect (E_max_) of 200% (95% C.I. 127–273%). In contrast, CL significantly compressed this curve cutting the maximum almost into halves (E_max_ = 110%, 95% C.I. 71–149%; Fig. 2c and Table 1; *P*<0.01, two-way-ANOVA with post-hoc Tukey test), but without altering EC_50_ (0.8 μM). Hence, although CL did not affect KCl responses in this patient cohort, CCh-induced contractions proved to be responsive to this compound (Cohen’s d of 1.82 in mid-life versus 0.13 in aged subjects). In aged humans, however, both CCh CRCs (i.e. with and without CL) were rather comparable (Fig. 2d, Table 1). Taken together, β_3_ adrenoceptor activation by CL reduced the potency of CCh to evoke contractions only in the mid-life sub-cohort, but not the aged. Moreover, CL left the EC_50_ unaltered implying a non-competitive mechanism of action.

**Table 1:** Inhibition of carbachol-induced contraction by CL316,243 in human and rat detrusor. Numbers in parentheses indicate 95% confidence interval

	Carbachol CRC	CL316,243 conc.	Adolescent rats *or* mid-life humans	Mid-life rats *or* aged humans
Rat			<65 days	≥65 days
	EC_50_	None	1 μM	1 μM
	E_max_	None	160% (32–288%)	130% (105–155%)
	EC_50_	0.1 μM	1 μM	0.8 μM
	E_max_	0.1 μM	130% (23–237%)	120% (80–160%)
	EC_50_	1 μM	1 μM	0.8 μM
	E_max_	1 μM	120% (0–305%)	120% (98–142%)
	EC_50_	10 μM	1 μM	0.8 μM
	E_max_	10 μM	100% (63–137%)	120% (81–159%)
Human			<65 years	≥65 years
	EC_50_	None	0.8 μM	0.8 μM
	E_max_	None	200% (127–273%)	240% (189–291%)
	EC_50_	10 μM	0.8 μM	0.8 μM
	E_max_	10 μM	110% (71–149%)	230% (113–347%)

### The β_3_ agonist CL316,243 dampens rat bladder contraction in an age-dependent manner

**Fig. 3:**
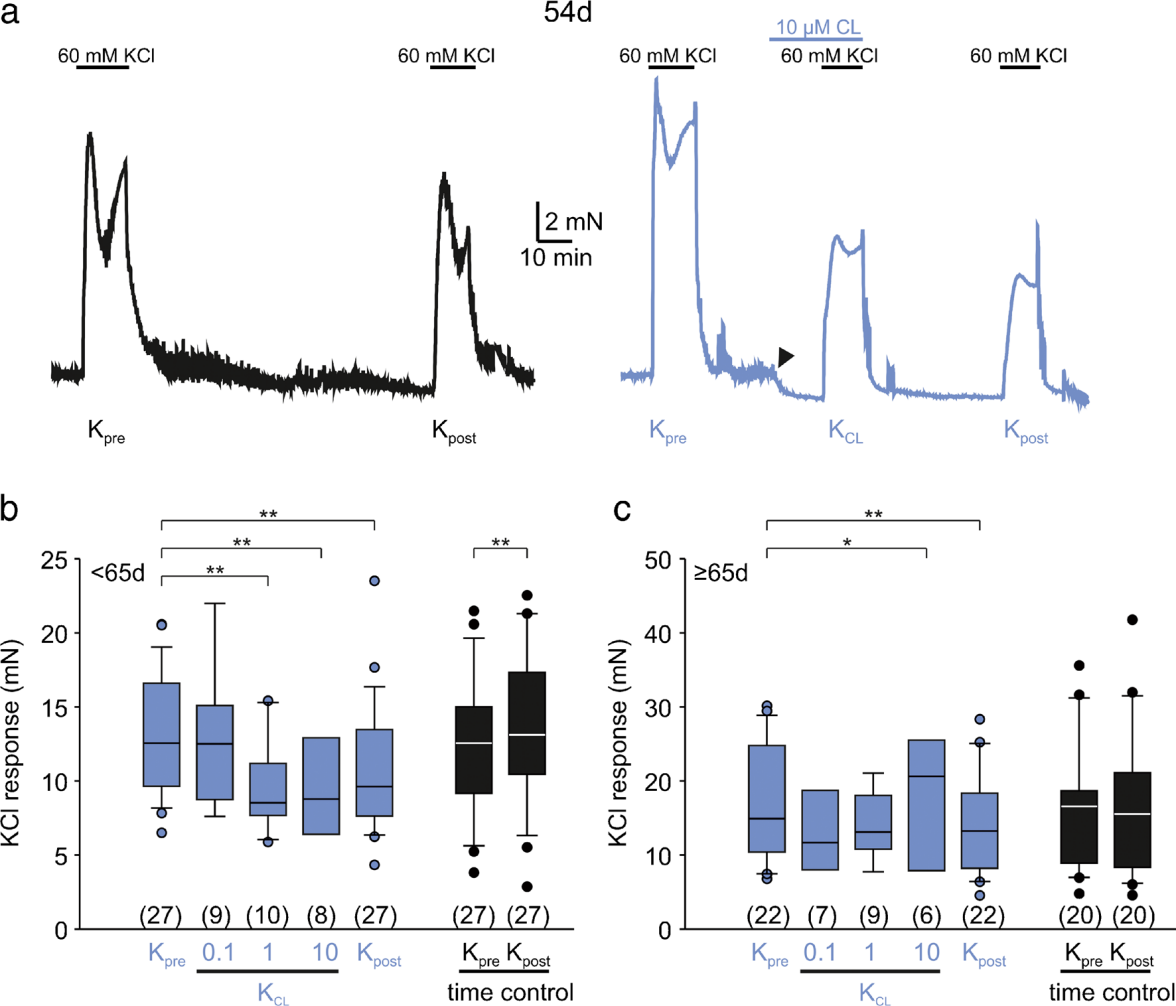
CL316,243 inhibits KCl-induced contraction in rat detrusor. **a** Representative traces of KCl-induced contractions in a 54-day-old rat from a time-control experiment (black) and a CL testing experiment (blue). Note the drop of the baseline tone after CL application (arrowhead). **b** CL dose-dependently reduced the subsequent CCh-induced contractions in animals <65 days (Wilcoxon signed rank test). All *n*-numbers refer to animals. **c** CL had a statistically significant effect on KCl-induced contractions in animals ≥65 days only at 10 μM

Having established a differential responsiveness of human detrusor tissue towards β_3_ adrenoceptor activation, we wished to ascertain whether rat tissue confirmed these results. Figure 3 illustrates the influence of CL on rat bladder KCl-induced contractions. Whereas successive applications of 60 mM KCl in time control experiments elicited reproducible results in a 54-day-old animal (black trace in Fig. 3a), CL markedly reduced the KCl response in an adjacent bladder specimen, and moreover, entailed poor washout (blue trace in Fig. 3a). Moreover, CL effectuated a statistically significant drop of baseline tone (−8 ± 8% at 10 μM, mean ± SD; see arrowhead in the blue trace in Fig. 3a). Like the human tissue study, we divided our animal cohort (from 40 days to 1.5 years of age) into two sub-cohorts of about the same sample size, here either below 65 days (referred to as adolescent) or above (referred to as mid-life). In 106 specimens from 27 adolescent rats, we found a statistically significant and dose-dependent attenuating effect of CL on KCl-induced contraction (*P*<0.01, Wilcoxon signed rank test, blue box-whisker plots in Fig. 3b), while time controls showed a statistically significant increase (*P*<0.01, Wilcoxon signed rank test, black box-whisker plots in Fig. 3b). In contrast, CL affected the KCl response in the mid-life group only at the highest concentration (Fig. 3c). Since the absolute KCl response depends on parameters such as strip size or orientation of smooth muscle cells within the specimens, we wished to control for this and separately calculated *K*_pre_ and *K*_post_ for each CL subgroup. These analyses showed that all averaged amplitudes were quite comparable (Table 2). Two-way ANOVA (factors age, and CL subgroup) on *K*_pre_ revealed a significant age effect (*P*<0.05), but no significant CL effect (*P*=0.392). *K*_pre_ was 13.3 ± 3.8 mN (*n*=27) in adolescent rats and 16.7 ± 7.6 mN (*n*=22) in mid-life rats. We infer from these data that specimen preparation was quite homogenous within the same age group, but there was a statistically significant difference between adolescent and mid-life rats.

**Table 2:** Absolute KCl-induced contraction forces (mean ± SD) in all rat CL subgroups (according to Fig. 3b, c)

	<65 days		≥65 days	
CL subgroup	*K*_pre_ (mN)	*K*_post_ (mN)	*K*_pre_ (mN)	*K*_post_ (mN)
0.1 μM	15.1 ± 4.1 (*n*=9)	13.2 ± 4.5 (*n*=9)	13.8 ± 5.4 (*n*=7)	13.4 ± 7.1 (*n*=7)
1 μM	12.4 ± 3.3 (*n*=10)	9.5 ± 2.3 (*n*=10)	15.9 ± 6.3 (*n*=9)	13.1 ± 4.1 (*n*=9)
10 μM	12.3 ± 3.5 (*n*=8)	9.3 ± 4.1 (*n*=8)	21.4 ± 9.1 (*n*=6)	16.8 ± 7.4 (*n*=6)

**Fig. 4:**
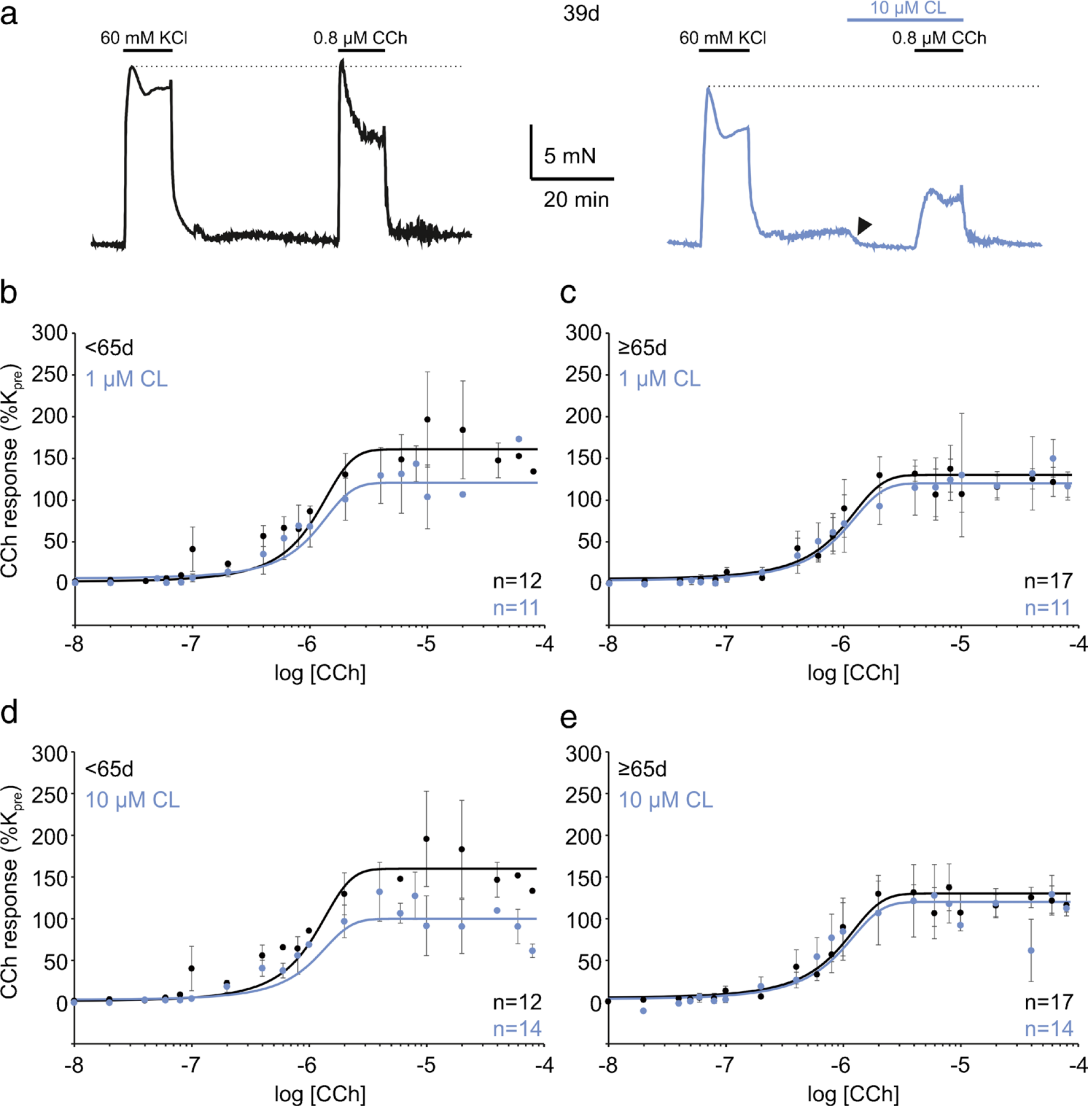
CL316,243 inhibits CCh-induced contraction in rat detrusor. **a** CCh-induced contractions were normalised to KCl-induced contractions (indicated by dotted lines). In a 39-day-old rat, the CCh response was almost as large as the KCl response (black), but administration of CL (blue traces) markedly reduced the CCh response in comparison to the KCl response. **b**–**e** Concentration-response curves with (blue) and without (black) prior CL application. A significant CL effect was obtained with 1 μM (**b**) and 10 μM (**d**), but not in animals ≥65 days (**c** and **e**). Data are presented as mean ± SD, all *n*-numbers refer to animals

We then set out to assess the impact of CL on CCh responses in the rat bladder. Here, in a bladder specimen from a 39-day-old animal, 60 mM KCl and 0.8 μM CCh produced contractions of comparable size (black traces in Fig. 4a). When, however, CL was present prior to CCh administration, the 0.8 μM CCh response was half as large as the 60 mM KCl response (blue traces in Fig. 4a). To study the CL effect on the CCh response systematically, we randomly elicited the CCh response in 96 animals to obtain CRCs with or without CL pre-treatment (0.1, 1, and 10 μM). After dividing our animal cohort into two sub-cohorts (as above), the two-way-ANOVA followed by Tukey post-hoc test revealed a significant treatment effect of all three CL concentrations tested in the adolescent group (all *F* values >8 and *P*<0.01 for 0.1, 1, and 10 μM), but not the mid-life group (Table 1 and Fig. 4b–e). Importantly, the EC_50_ remained almost constant pointing again to a non-competitive mechanism of CL in inhibiting the CCh response. In summary, adolescent but not mid-life rat bladder specimens consistently responded to CL with reduced KCl and CCh responses.

### Immunohistochemical detection of β_3_ adrenoceptors in human detrusor

**Fig. 5:**
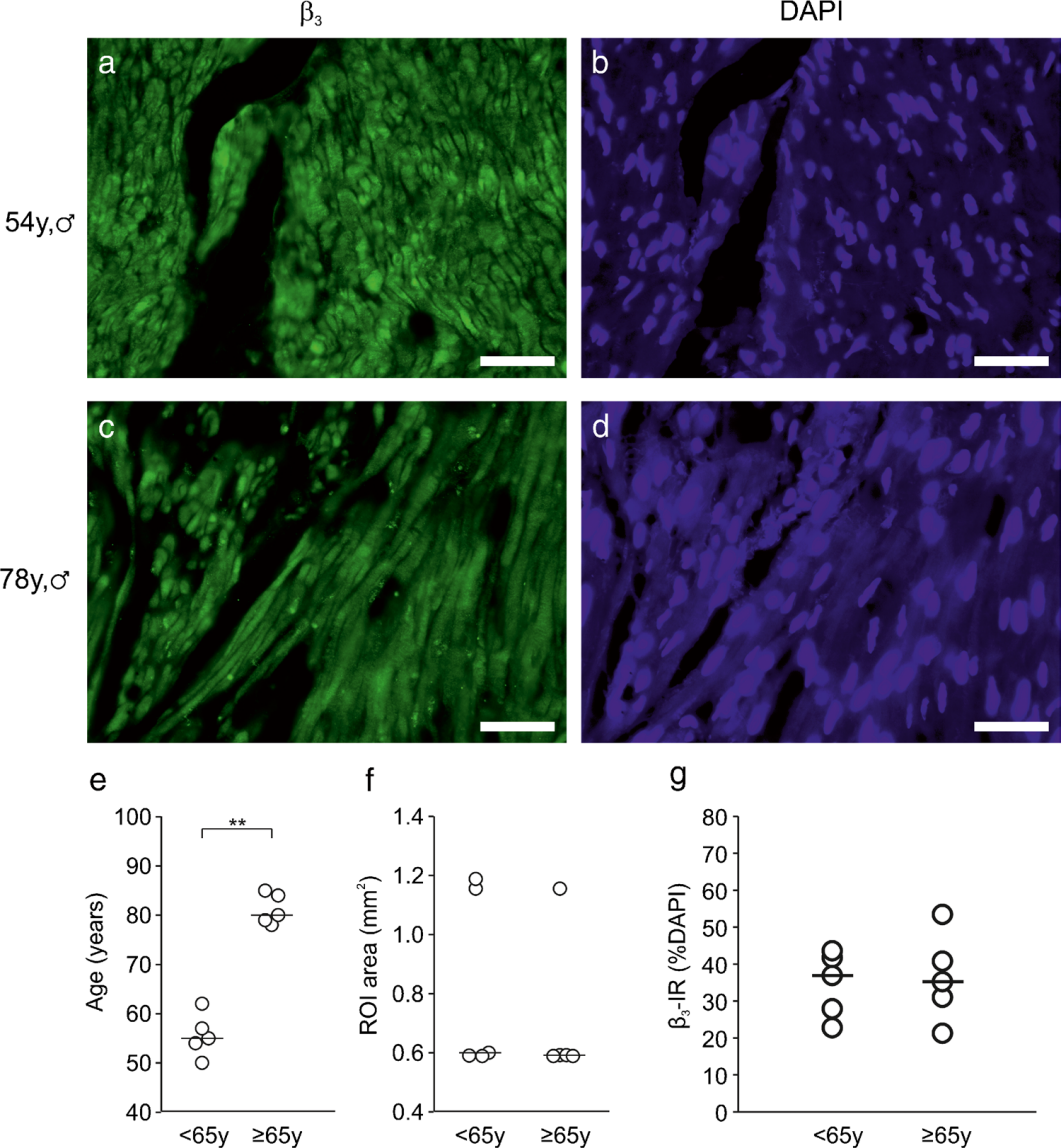
Immunohistochemistry of β_3_ adrenoceptors in human detrusor. **a**–**d** β_3_ immunoreactivity in human detrusor from a 54-year-old patient (**a**) and a 78-year-old patient (**c**). Note the difference in staining which resembles the fainter nuclear staining with DAPI in the older patient (**d**) compared with the younger one (**b**). Scale bar = 50 μm. **e**–**f** Analysis of two different age groups (*P*<0.01, Mann-Whitney *U* test) (**e**) with similar regions of interest (**f**) showed similar β_3_ immunoreactivity when normalised to DAPI (**f**). *n*-numbers refer to patients

We next wondered whether β_3_ adrenoceptor expression levels maintain with age. To this end, we carried out immunohistochemistry in human sections from ten patients (nine males, one female) with previously used anti-β_3_ antibodies [46]. Detecting β_3_ immunoreactivity throughout the detrusor smooth muscle in all ten patients tested (Fig. 5a–d), we observed a somewhat lesser staining in sections from aged patients (Fig. 5c). However, nuclear staining with DAPI was also diminished in this cohort indicative of a loss of nuclei (Fig. 5d). To discern a difference between mid-life and aged tissue samples, we analysed one group with a mean age of around 55 years and another one with a mean age of around 80 years with comparable regions of interest (Fig. 5e, f). Normalised to DAPI nuclear stain as a measure of cell density, we failed to find a statistically significant difference between both groups (Fig. 5g). From these data, we cannot infer a decline in β_3_ adrenoceptor abundance during ageing in males.

## Discussion

The present study aimed at elucidating the age-dependent role of β_3_ adrenoceptor-mediated attenuation of urinary bladder contraction in human and rat tissue. We found that high KCl and carbachol (CCh) evoked tonic contractions in human detrusor muscle strips, which were differentially affected by prior β_3_ adrenoceptor activation with the selective agonist CL316,243 (CL). Whereas the KCl responsiveness was greater in tissue from aged patients, the CCh responsiveness was greater in tissue from mid-life patients. In contrast, tissue from adolescent rats was generally more sensitive to CL than tissue from mid-life rats. Immunohistochemistry confirmed the presence of β_3_ adrenoceptors on human detrusor smooth muscle cells but failed to detect a difference in receptor abundance between mid-life and aged tissue.

### Role of β_3_ adrenoceptors in resting detrusor

One major finding of our study was that the β_3_-specific agonist CL reduced both basal tone and spontaneous contractions. This corroborates previous reports on non-selective β agonists such as isoprenaline [22, 23], as well as β_3_-selective agents showing higher potencies than β_1_ or β_2_ agents such as dobutamine, clenbuterol, or procaterol [5, 23, 57]. Although not specifically addressed, age-dependent changes seem rather unlikely in all these studies, as they included tissue samples from 5- to 82-year-old patients. Since we did not use β_3_ adrenoceptor antagonists, we cannot exclude β_3_-independent effects on resting detrusor. Based on our data on age-dependent changes in CL sensitivity, we infer that β_3_-mediated relaxation of resting human detrusor was irrespective of the donor’s age.

With respect to intracellular signalling, β adrenoceptors couple positively to adenylate cyclase which by itself promotes detrusor relaxation [10]. In theory, this would directly counteract negative coupling to this enzyme through M_2_ receptors. From experimental animals, we know that both β_3_ adrenoceptors and M_2_ receptors are expressed on smooth muscle cells to provide a functional antagonism [20, 21, 29, 30, 35, 43, 44, 49, 58]. Whilst there is evidence that in human detrusor, M_3_ receptors, although less available, outweigh the M_2_-mediated contraction [11], some M_2_-dependent tone may be presumed which would imply acetylcholine release, e.g. arising from nerve sources during urothelial stretching [47]. Albeit being of minor relevance in humans, we infer from our data that an M_2_-dependent basal tone also exists in humans and that β_3_ activation may increase both compliance and capacity as demonstrated in rats [54].

We found no evidence for an age-dependent decline in β_3_ adrenoceptor abundance in human detrusor. It is well established that β_3_ adrenoceptors are expressed on human detrusor smooth muscle; its mRNA substantially outreaches—by far more than 90%—the β_1_ and β_2_ isoforms [20, 22, 27, 39, 50, 51, 56]. Several immunohistochemical studies confirmed β_3_ protein being located on these cells [28, 40, 46]. One major drawback of immunohistochemical studies on β adrenoceptors is target specificity [41]. Here, we chose the antibody AAR-017 [46] because it was raised against the human and rat receptor, and it showed the same results as the validated antibody MC-A4198 [28, 40, 46]. Although the patients’ ages ranged from 18 to 77 years in these studies, age dependence was rarely analysed, except for one study where β_3_ adrenoceptor expression remained stable, at least in a small female cohort [28]. Consistent with this study, we found comparable β_3_ staining in mid-life and aged tissue—while having a clear male preponderance in our sample. Hence, the present study not only confirms available data, but also adds to the literature by demonstrating maintained β_3_ expression in the elderly male.

### Role of β_3_ adrenoceptors in KCl-induced detrusor contraction

The effects of β_3_ adrenoceptors on resting detrusor do not necessarily predict to what extent externally evoked contractions are concerned. KCl-induced contractions were slightly more attenuated by CL in aged tissue than in mid-life tissue, whereas CCh-induced contractions showed higher CL sensitivity in mid-life than in aged tissue. This implies that different contraction mechanisms differ in their β_3_ responsiveness, as previously suggested [7]. In particular, KCl depolarises the cell membrane and leads to contraction by Ca^2+^ influx through voltage-gated Ca^2+^ channels (reviewed by [34]). CCh, in turn, activates the muscarinic receptor and leads to contraction by Ca^2+^ release from internal stores or by Ca^2+^ sensitisation through G_q/11_ and G_12/13_ coupling, respectively (reviewed by [55]). Although cross-talk may exist between these pathways to a certain extent, differential β_3_-mediated effects occurring during ageing point to interference among intracellular signalling cascades rather than to age-dependent changes in receptor abundance. In the following paragraphs, we will discuss KCl-induced contractions; CCh-induced contractions will be discussed thereafter.

Almost all studies so far analysed β_3_ effects on KCl-precontracted detrusor and largely disregarded the impact of age, which impedes reconciling their findings with our own data. At least, the maximally achieved effects were comparable with ours, even though with relatively high concentrations: CL or BRL37244 (both at 100 μM) relieved the KCl-induced contraction by around 35% in a mid-life cohort [2, 3]. Others normalised the KCl response relieving effect to broad-spectrum relaxants such as isoprenaline or papaverine and obtained conspicuously higher maximum effects ranging from 56% to almost 100% [27, 51]. One of these even claimed a significantly reduced effect in aged tissue as opposed to young adult “control” tissue [27]. However, this was certainly due to (1) reduced isoprenaline effects in the aged tissue, and (2) missing time control experiments [27], and thus awaits further confirmation. In view of our data, we draw the conclusion that β_3_-mediated inhibition of KCl-induced contractions is at least preserved during ageing.

In contrast to humans, rats showed reduced CL attenuating effects of KCl-induced contractions during ageing. Nishimoto and co-workers had similarly observed that aged rat bladder was less sensitive to isoproterenol, forskolin, or cholera toxin [38], which was confirmed using β_3_-selective agonists [18]. To date, it is not clear whether species differences in intracellular signalling cascades are responsible for the differential CL effects. At least, three aspects need to be considered: (1) G protein abundance in rat bladder is affected by age [14], (2) the Rho kinase inhibitor blocks CCh-induced contractions by ~45% in humans [7, 25], compared with ~60% in rats [7], and (3) the combined inhibition of Rho kinase and myosin light-chain kinase fails to abolish contraction in humans [25]. When comparing human and rat experiments, one should also take into consideration that the investigated ages were not overlapping and that experimental designs were not identical in both species. While the mucosa has been removed from human bladder strips, it was intact in bladder strips. We thus cannot fully exclude mucosa-mediated effects in rat specimens. Therefore, we can only speculate that the human detrusor exhibits multiple parallel contraction pathways, which can compensate for each other in case of a shortage of one of these, e.g. during ageing.

### Role of β_3_ adrenoceptors in CCh-induced detrusor contraction

In contrast to KCl, our study demonstrated an age-dependent decline of β_3_-mediated inhibition of CCh-induced contractions in human and rat detrusor samples. Except for mirabegron, previous studies in humans revealed submicromolar to low micromolar sensitivity of β_3_ agonists, which was compatible with our EC_50_ of ~1 μM [5, 23, 39, 50]. Age dependence, however, was not addressed in these studies: analysing 50 patients from 5 to 82 years, Igawa and co-workers observed stronger CL-mediated relaxation in normal than in hyperreflexic detrusor, but without acknowledging the patient’s age as a relevant covariate [23]. The remainder could not address this issue owing to their small sample sizes (12 patients aged from 60 to 68 years in [50]; seven patients aged from 53 to 76 years in [39]; 12 patients aged from 28 to 75 years in [5]). To what extent different experimental paradigms in human studies affect age dependence needs to be clarified. Taken together, we confirmed this age-dependent decline in rat tissue, consistent with (1) functional β_3_ adrenoceptor expression on rat detrusor [20, 30, 44] and (2) reduced adenylate cyclase activity in aged rats [53], probably due to a differential change in G protein abundance [14]. Future studies will need to address whether these issues also hold for human detrusor.

What is the clinical implication of our study? A recent review clarified that anticholinergics and β_3_ agonists showed similar efficacy, but tolerability issues clearly favoured the latter [31]. Since OAB is more prevalent in the elderly, reduced β_3_ responsiveness of bladder smooth muscle cells could be of clinical interest, even though—at present—there is little evidence for this [36]. As a matter of fact, direct detrusor relaxation by β_3_ agonists is only one mechanism of action, while others such as β_3_ adrenoceptor-mediated effects on afferent nerve fibres [12] may preserve their function during aging. Our data demonstrated a relevant difference in β_3_ sensitivity of electromechanical versus pharmacomechanical coupling. While the first is maintained in the elderly, the latter is reduced. This will become particularly relevant as far as both ionotropic and metabotropic receptors of the same transmitter are concerned, e.g. in the purinergic system [17, 19]. Conceivably, this will also help understand the responsiveness of OAB patients to β_3_ agents.

## Data Availability

Data will be made available on request.
